# Molecular Characterization of Tropomyosin and Its Potential Involvement in Muscle Contraction in Pacific Abalone

**DOI:** 10.3390/genes14010002

**Published:** 2022-12-20

**Authors:** Md Abu Hanif, Shaharior Hossen, Won Kyo Lee, Kang Hee Kho

**Affiliations:** Department of Fisheries Science, Chonnam National University, Yeosu 59626, Republic of Korea

**Keywords:** cloning, gene ontology, coiled-coil structure, expression analysis, contractile function

## Abstract

Tropomyosin (TPM) is a contractile protein responsible for muscle contraction through its actin-binding activity. The complete sequence of *TPM* in *Haliotis discus hannai* (*Hdh-TPM*) was 2160 bp, encoding 284 amino acids, and contained a TPM signature motif and a TPM domain. Gene ontology (GO) analysis based on the amino acid sequence predicted Hdh-TPM to have an actin-binding function in the cytoskeleton. The 3D analysis predicted the Hdh-TPM to have a coiled-coil α-helical structure. Phylogenetically, Hdh-TPM formed a cluster with other TPM/TPM1 proteins during analysis. The tissue-specific mRNA expression analysis found the higher expression of *Hdh-TPM* in the heart and muscles; however, during embryonic and larval development (ELD), the higher expression was found in the trochophore larvae and veliger larvae. *Hdh-TPM* expression was upregulated in fast-growing abalone. Increasing thermal stress over a long period decreased *Hdh-TPM* expression. Long-term starvation (>1 week) reduced the mRNA expression of *Hdh-TPM* in muscle; however, the mRNA expression of *Hdh-TPM* was significantly higher in the mantle, which may indicate overexpression. This study is the first comprehensive study to characterize the *Hdh-TPM* gene in Pacific abalone and to report the expression of *Hdh-TPM* in different organs, and during ELD, different growth patterns, thermal stress, seasonal changes, and starvation.

## 1. Introduction

Tropomyosin (TPM), a dimeric-helical coiled-coil protein [1], is thought to be the master regulator of actin filament functions in the cytoskeleton [2] since it regulates actomyosin interactions, which are a central feature of contractile events in animal cells [3]. Actomyosin interactions are controlled by the actin-binding proteins TPM and troponin [4] through a Ca^2+^-dependent switch mechanism [5]. During contraction, the sarcomeres of striated muscles and actin and myosin filaments are arranged in parallel and slide past each other to cause sarcomere shortening. When Ca^2+^ binds to troponin, TPM moves azimuthally on the actin filament, exposing the myosin-binding site. Myosin attaches to the actin filament and performs a power stroke, which results in the shortening of the sarcomere. During relaxation, as Ca^2+^ levels drop, this process is reversed [6]. At low Ca^2+^ concentrations, troponin locks TPM in position on the F-actin, where it obstructs the myosin-binding site, thus preventing the contraction of the sarcomere [7]. During muscle contraction, skeletal muscles secret decorin, which directly binds to and inactivates myostatin (a potent inhibitor of muscle growth) and thus promotes muscle growth [5]. Muscle contraction is one of the important physiological processes regulated by TPM through control of actomyosin interactions [8]. Contractions appear to upregulate muscle cell activity and anabolic signaling pathways to a greater extent [9]. Muscle mass increases during postnatal development through a process of hypertrophy in response to contractile activity [10].

In invertebrates, TPM has been reported to occur during embryonic and larval development (ELD), indicating that TPM may play an important role in ELD and adult life [11]. However, the function of TPM in the ELD was unknown until now. In invertebrates, such as nematodes, RNA interference of TPM caused sterility by inhibiting the ovarian contractions required to ejaculate fully-grown oocytes into the spermatheca where fertilization occurs, thus causing an accumulation of endomitotic oocytes in the ovary [12].

The temperature dependence of physiological processes in marine ectotherms is well recognized [13]. The heat released at the time of muscle contractions naturally increases body and tissue temperatures [14]. However, elevated temperatures are known to result in an early onset of fatigue [15]. The elevated temperatures can cause protein unfolding, denaturation, and aggregation, which can impair muscle contractile protein functions and hamper muscle growth [16]. These activities induce physiological and behavioral responses that enable the individual to cope with challenges [17]. Physical and behavioral changes are common in aquatic animals, depending on the season, since seasonal changes are reflected in temperature changes. For instance, abalone stops feeding at temperatures below 5 °C and reduces feeding at temperatures above 20 °C, resulting in reduced physiological functions due to an insufficient supply of micronutrients from food [18]. During this starvation period, the functionality of many genes is altered, including TPM, which regulates muscle contraction.

Abalones are a group of marine gastropods that belong to the Haliotidae family. Approximately 56 abalone species are found in the temperate and tropical coastal habitats of the United States, Australia, and East Asia [19,20]; however, most abalones inhibit temperate waters. Due to high market demand, natural abalones have undergone intensive fishing worldwide, resulting in stock depletion in most countries [21]. Thus, the mariculture of abalone was established and has largely expanded since the 1970s to reduce the fishing of natural abalone populations and to increase the annual production [22]. However, the abalone aquaculture mortality rate is increasing due to heat stress caused by global warming, especially in the summer season. The Pacific abalone, *H. discus hannai*, is extensively cultured among the available abalone species due to its high value and commercial importance not only in South Korea but in many Asian countries [23,24,25,26]. Since the foot muscle is the main edible portion of the abalone [27], the growth and development of this muscle is a vital issue in abalone aquaculture and is mainly dependent on muscle growth and contraction regulating genes. Generally, muscle growth, categorized as a heritable feature, can be modulated by fiber type position, metabolism, contractile speed, temperature, and food availability [28]. In this study, the muscle contraction regulatory gene *Hdh-TPM* was isolated and characterized from Pacific abalone muscle tissue, and the changes in mRNA expression were documented in different developmental stages, growth modes, and experimental conditions.

## 2. Materials and Methods

### 2.1. Experimental Animal and Sample Collection

Three-year-old sexually mature male and female Pacific abalones with a mean body weight of 120.4 ± 0.61 g and a mean shell length of 84.06 ± 0.32 mm were collected from sea cages from Jindo-gun, South Korea. The collected Pacific abalones were transported using an icebox to the molecular physiology laboratory at the Department of Fisheries Science, Chonnam National University, Gwangju, South Korea.

### 2.2. Tissues Collection for Gene Cloning and Expression Analysis

Prior to tissue sample collection, abalones were subjected to anesthetization using 5% MgCl_2_. For cloning, a total of 12 Pacific abalones were sacrificed, and their hemocyte, cerebral ganglion, pleuropedal ganglion, testis, ovary, mantle, muscle, gill, heart, and digestive gland tissues were collected. The collected tissue samples were washed with phosphate-buffered saline (PBS; 0.1 M), immediately snap-frozen in liquid nitrogen, and stored at −80 °C until total RNA extraction.

### 2.3. Fertilization and Sample Collection from the Embryonic and Larval Development (ELD) Stages

Artificial fertilization of Pacific abalones was performed following the procedure described by Hanif et al. [29]. Briefly, male abalones with fully mature gonad were induced to release sperm by sunlight exposure (60 min shell-side down and 40 min shell-side down). Afterward, male and female abalones were induced separately using ultraviolet-treated seawater. Immediately after spawning, sperm and eggs were collected and fertilization was conducted in filtered seawater, maintaining a water temperature of 18 °C. Maintaining a ratio of 1:10,000 (egg: sperm), approximately 30,000 eggs were mixed with the required amount of sperm. Ten minutes later, the fertilized eggs were washed three times with filtered seawater to remove the oil layer. Through subtle observation under a microscope, the samples of target stages, including fertilized eggs, two-cell and four-cell, blastula, trochophore larvae, veliger larvae, and juveniles, were collected from three different replicates in 1.5 mL microtubes and immediately snap-frozen in liquid nitrogen and stored at −80 °C until total RNA extraction. In each microtube, there were nearly 1000 embryos or larvae collected from each stage.

### 2.4. Tissue Samples from Heat-Stress-Treated Pacific Abalones

To observe the stress response of *Hdh-TPM* in Pacific abalones, a heat treatment was performed. Adult Pacific abalones (shell length: 83 ± 3.7 mm, shell width: 42 ± 2.9 mm, and total weight: 122 ± 1.6 g) were purchased from an abalone farm in Jindo-gun, South Korea and were transported to the abalone hatchery in Yeosu, South Korea. The abalones were reared in culture tanks with a maintained water temperature of 20.4 ± 0.3 °C for two weeks to allow recovery from the stress of transportation. The abalones were then divided into three groups and kept for a 48 h acclimation period in 15 °C, 25 °C, and 30 °C seawater controlled by a digital temperature controller. No food was provided during this heat-stress treatment. Tissue samples from the muscle and mantle were collected after 1, 6, 12, 24, and 48 h and were washed with 0.1 M PBS. After washing, the tissues were immediately snap-frozen in liquid nitrogen and stored at −80 °C until total RNA extraction. Initial control samples were also collected and stored following this process.

### 2.5. Tissue Samples from Different Growth Types of Pacific Abalone

Three-year-old fast-growth and slow-growth Pacific abalones were collected from the culture area. A total of ten abalones of each growth type were anesthetized with 5% MgCl_2_ and sacrificed for sample collection. Mantle and muscle tissues were collected, washed with 0.1 M PBS, and stored at −80 °C after being snap-frozen in liquid nitrogen.

### 2.6. Tissue Samples from Starved Pacific Abalones

Three-year-old Pacific abalone having a body weight of 100.2 ± 0.56 g and a mean shell length of 80.03 ± 0.31 mm were randomly collected from the sea cages in Jindo-gun, South Korea, and transported to the abalone hatchery in Yeosu, South Korea. The collected abalones were reared in rectangular cemented tanks with running seawater flow and adequate feed supply for an adjustment period. After a month, tissue from the mantle and muscle of ten Pacific abalones was collected, following the procedure mentioned above, and stored for total RNA extraction. Additional abalones were reared without feeding, and tissue samples (mantle and muscle) were collected every seven days for three weeks and seven days after refeeding, following the procedure mentioned above, and stored at −80 °C until total RNA extraction.

### 2.7. Seasonal Tissue Sample Collection from Pacific Abalones

Three-year-old Pacific abalones were collected seasonally (winter, spring, autumn, and summer) and sacrificed after anesthetizing with 5% MgCl_2_. Mantle and muscle tissues were collected and washed with 0.1 M PBS. After washing, the tissues were immediately snap-frozen in liquid nitrogen and stored at −80 °C until total RNA extraction.

### 2.8. Extraction of RNA and cDNA Synthesis

Total cellular RNA was extracted from the collected tissue samples using an ISOSPIN Cell and Tissue RNA kit (Nippon Gene, Tokyo, Japan). First stand cDNA was synthesized from 1–4 μL of total RNA using oligo (dT) primers (OdT) (Sigma) and a superscript III First-strand cDNA synthesis kit (Invitrogen, Molecular Probes, Eugene, OR, USA). Using a SMARTer^®^ RACE 5′/3′ Kit (Takara Bio Inc., Kusatsu, Shiga, Japan), 5′- and 3′-RACE cDNA was synthesized from 1 μL of tRNA from muscle tissue. Extraction of total RNA and cDNA synthesis was conducted following the manufacturer’s protocol.

### 2.9. Cloning of the Full-Length Tropomyosin Sequence in Pacific Abalone

#### 2.9.1. Partial Sequence Cloning

For the partial sequence cloning of *Hdh-TPM*, a reverse transcription polymerase chain reaction (RT-PCR) was conducted using cDNA from the muscle tissue, a set of primers (forward and reverse), and GoTaq^®^ DNA Polymerase (Promega, Madison, WI, USA). The primer set used in this study was designed from the *TPM* nucleotide sequence from *H. discus discus* (GenBank Accession no. AB444939.1). All primers used in this experiment are presented in Table 1. The reaction mixture for the RT-PCR was prepared to a total volume of 50 μL and contained cDNA (1 μL), 20 pmol forward (*Hdh-TPM* RT Fw) and reverse (*Hdh-TPM* RT Rv) primers (1 μL each), GoTaq reaction buffer (10 μL), dNTP mix (1 μL), DNA polymerase (0.25 μL), and ultrapure water (35.75 μL). The RT-PCR thermal cycling condition was as follows: initial denaturation for 3 min at 95 °C; followed by 35 cycles of denaturation for 30 s at 95 °C, annealing for 30 s at 58 °C, and extension for 45 s at 72 °C; and a final extension for 5 min at 72 °C. Obtained PCR products were subjected to 1.2% agarose gel electrophoresis, and positive band was purified using a Wizard^®^ SV Gel and PCR Clean-Up System kit (Promega, Madison, WI, USA). The purified DNA was then ligated into the pGEM^®^-T Easy Vector (Promega, Medison, WI, USA) and transformed into DH5α chemically competent *Escherichia coli* (Enzynomics, Daejeon, Republic of Korea). The positive clones were selected for plasmid DNA purification using a Hybrid-QTM Plasmid Rapidprep mini kit (GeneAll, Seoul, Republic of Korea) and were sequenced at Macrogen (Seoul, Republic of Korea).

#### 2.9.2. RACE (5′- and 3′-) Sequence Cloning

To obtain the full-length sequence of *Hdh-TPM*, a rapid amplification of cDNA ends polymerase chain reaction (RACE-PCR) was performed using a SMARTer^®^ RACE 5′/3′ kit (Takara Bio Inc., Japan). A set of gene-specific 5′- and 3′-RACE primers were prepared from the obtained partial sequence of *Hdh-TPM*, including a 15 bp (GATTACGCCAAGCTT) overlap at the 5′-end of the primer sequence. The 5′- and 3′-RACE-PCRs were performed using 2.5 μL of 3′- or 5′-RACE cDNA, 1 μL of sense (3′RACE) or antisense (5′RACE) RACE primers, 5 μL of a universal primer mix (UPM), 1 μL of SeqAmp DNA polymerase, 25 μL of SeqAmp buffer, and 15.5 μL of PCR-grade water. Touchdown PCR was carried out with 30 cycles for both 3′-RACE and 5′-RACE. The thermal cycle conditions for the RACE-PCR were maintained according to the conditions prescribed in the kit. The PCR products were subjected to gel electrophoresis (as described previously), and purification was performed using a NucleoSpin^®^ Gel and PCR Clean-up kit (MACHEREY-NAGEL GmbH & Co., KG, Dueren, Germany). The purified products were ligated into a linearized pRACE vector and transformed into stellar competent cells. Positive clones were purified and sequenced at Macrogen, as previously described, for the partial sequence. Finally, the 5′-RACE sequence, the initially cloned partial cDNA fragment, and the 3′-RACE sequence were combined and trimmed to obtain the full-length sequence.

### 2.10. Sequence Analysis of Cloned H. discus hannai Tropomyosin (Hdh-TPM)

The complete nucleotide and protein sequence of the cloned *Hdh-TPM* were analyzed by using several online tools. Potential protein encoding segments and open reading frames (ORFs) were predicted from the nucleotide sequence using the online tool ORFfinder (https://www.ncbi.nlm.nih.gov/orffinder/; accessed on 23 August 2022). The molecular weight of Hdh-TPM protein and the isoelectric point (pI) were computed using the online tool ProtParam (https://web.expasy.org/protparam/; accessed on 23 August 2022). The protein structure and gene ontology of the Hdh-TPM protein were predicted using the online protein structure prediction server Contact-guided Iterative Threading ASSEmbly Refinement (C-I-TASSER) (https://zhanggroup.org/C-I-TASSER/; accessed on 17 July 2022). The functional domains of the Hdh-TPM protein were ascertained using the SMART server (http://smart.embl-heidelberg.de/; accessed on 6 August 2022) and the Motif scan (https://myhits.sib.swiss/cgi-bin/motif_scan; accessed on 6 August 2022). The conserved motifs of the Hdh-TPM protein were discovered using the Multiple Em for Motif Elicitation (MEME) online tool (v. 5.0.5; http://meme-suite.org/tools/meme; accessed on 13 September 2022). The coiled-coil region in the protein sequence was predicted using the online tool Waggawagga (https://waggawagga.motorprotein.de/; accessed on 18 August 2022). The multiple sequence alignment was performed using the MEGA version 11.0.13 software and visualized using Jalview version 2.11.1.7.

### 2.11. Orthology Analysis

The amino acid sequence from Hdh-TPM was aligned with the other related protein sequences using the online tool ClustalW. The phylogenetic tree was constructed and edited using the MEGA software (version 11.0.13) with a maximum likelihood algorithm.

### 2.12. Homology Modeling of Hdh-TPM

The predicted three-dimensional (3D) structure of Hdh-TPM was constructed using the previously mentioned protein structure and the functional prediction program, C-I-TASSER. The visualization of the 3D structure was performed using the UCSF ChimeraX software (v. 1.2.5).

### 2.13. Semiquantitative Reverse Transcription-Polymerase Chain Reaction (RT-PCR)

Semiquantitative RT-PCR was performed using gene-specific forward and reverse primers to observe the expression patterns of the *Hdh-TPM* in different tissues of Pacific abalone. A total of ten different tissues from Pacific abalones, including cerebral ganglion, pleuropedal ganglion, gill, testis, ovary, digestive gland, hemocyte, heart, muscle, and mantle, were used to observe the difference in *Hdh-TPM* expression. Due to the stable expression in different organs, *β-actin* from *H. discus hannai* (accession no. AY380809) was used as the internal control. A 20 μL reaction mixture was used, containing 1 μL of the cDNA template, 1 μL each of the forward and reverse primer, 10 μL of the 2× Prime Taq premix (GENETBIO, Daejeon, Republic of Korea), and 7 μL of ultrapure water. The RT-PCR thermal cycling conditions were maintained for the semiquantitative RT-PCR.

### 2.14. Quantitative Real-Time PCR (qRT-PCR) Analysis

To quantify the relative mRNA expression levels of *Hdh-TPM*, a quantitative real-time PCR (qRT-PCR) analysis was performed using different tissues from Pacific abalones. The expression levels of *Hdh-TPM* were observed in the different organs of adult Pacific abalone and the various embryonic and larval developmental stages of Pacific abalone. All qRT-PCRs were conducted following the protocol previously described by Hanif et al. [29]. In qRT-PCR analysis, three biological replicates were used in this study.

### 2.15. Statistical Analysis

The values of mRNA expression were analyzed statistically and expressed as the mean ± standard error. Changes in relative mRNA expression were computed using GraphPad Prism (version 9.3.1) software by a nonparametric ANOVA analysis. Statistical significance was set at a *p*-value less than 0.05. All graphs were prepared using MS Excel and GraphPad Prism 9.3.1 software.

## 3. Results

### 3.1. H. discus hannai Tropomyosin (Hdh-TPM) Sequence

**Figure 1:**
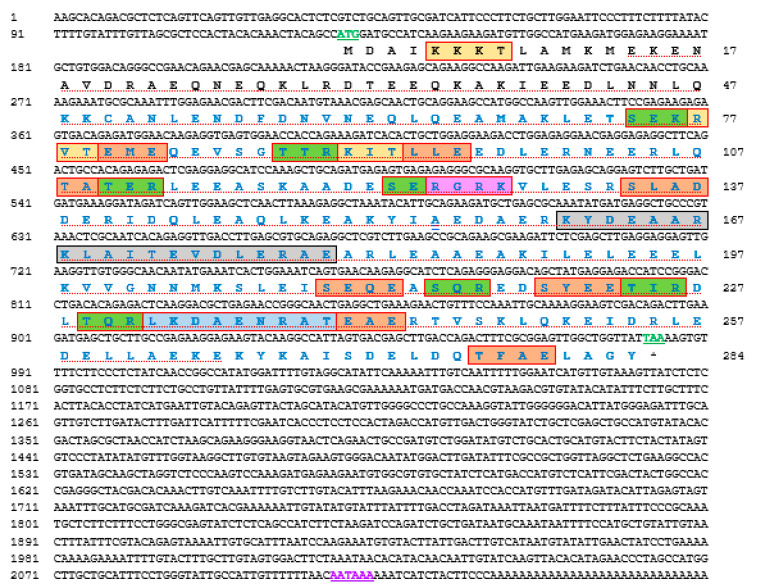
Full-length nucleotide and deduced amino acid sequences of *Hdh-TPM* (GenBank accession no. OM937906.1). The numerical numbers at the left and right side of the sequence indicate the position of nucleotide and amino acid of the *Hdh-TPM* gene, respectively. The coding region starts with a start codon (ATG) and ends with a stop codon (TAA), shown by green bold underline letter. The tropomyosin domain is marked with blue letters. The conserved tropomyosin signature peptide is marked with a light blue box. Cyclic adenosine monophosphate (cAMP) and cyclic guanosine monophosphate (cGMP)-dependent protein kinase phosphorylation sites are denoted by a yellow box. Potential protein kinase C phosphorylation sites are marked with a green box. Predicted casein kinase II phosphorylation sites are marked with an orange box. cAMP- and cGMP-dependent protein kinase phosphorylation sites are boxed in yellow. An amidation present in the sequence is shown with a pink box. The potential glutamic-acid-rich region profile is marked with a red dotted underline. N-terminal helix-turn-helix (HTH) site is indicated by an ash box. The putative polyadenylation signal is denoted by a violet letter with a plain underline.

The cDNA sequence encoding *Hdh-TPM* was cloned from the muscle tissue of *H. discus hannai* and designated as *Hdh-TPM*. The full-length sequence of the *Hdh-TPM* cDNA (GenBank accession OM937906.1) was 2160 bp long, including a poly-A tail (Figure 1). Its 5′- and 3′-untranslated regions (UTR) were 129 bp and 1176 bp long, respectively. A putative polyadenylation signal (AATAAA) was found in its nucleotide sequence at 46 bp upstream of the poly-A tail. The ORF of the *Hdh-TPM* cDNA sequence was 855 bp with 284 deduced amino acids.

The motif scan and conserved domain search suggested that Hdh-TPM had a TPM domain at the position of 48–284 amino acid residues with an E-value of 4.6 × 10^−120^. This protein also had a variation of the TPM signature sequence LKDAENRAT with a structure of L-K-[E/A/D]-A-E-x-R-A-[E/T] at the 232–240 amino acid position (Figure 1). A single amidation site X-G-[R/K]-[R/K] was found at the amino acid position 125–128 (RGRK) in the C-terminal region. A total of 18 phosphorylation sites were observed, of which seven were protein kinase C (PKC) phosphorylation sites, [S/T]-X-[R/K], at positions 74–76 (SEK), 88–90 (TTR), 110–112 (TER), 123–125 (SER), 215–217 (SQR), 224–226 (TIR), and 229–231 (TQR); eleven were casein kinase II phosphorylation sites, [S/T]-X(2)-[D/E], 79–82 (TEME), 93–96 (TLLE), 108–111 (TATE), 134–137 (SLAD), 172–175 (TEVD), 210–213 (SEQE), 215–218 (SQRE), 220–223 (SYEE), 224–227 (TIRD), 240–243, (TEAE), and 277–280, (TFAE); and three were cAMP- and cGMP-dependent protein kinase phosphorylation sites, [R/K](2)-X-[S/T], at position 5–8 (KKKT), 76–79 (KRVT), and 90–93 (RKIT). A spectrin repeat domain was observed at the position of 32–115 in the amino acid sequence.

**Figure 2:**
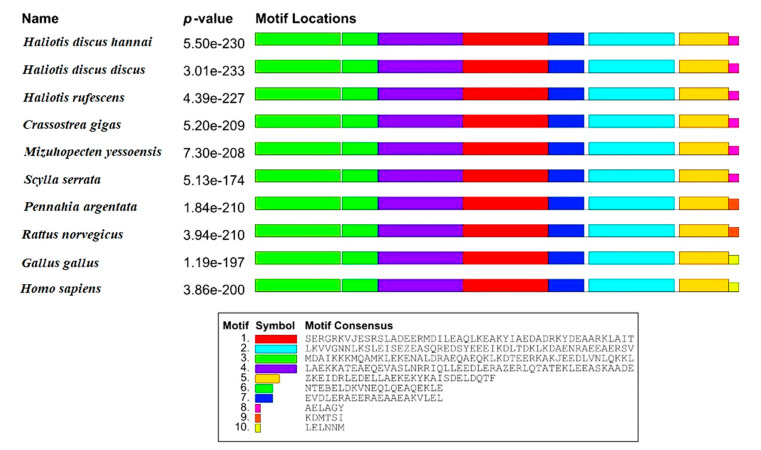
Schematic diagram of the motifs detected in Hdh-TPM and other TPM of invertebrates and vertebrates. Distinct motifs are denoted by different colors. The motif analysis included the TPM sequence of *H. discus hannai* and TPM sequences of other species: *H. discus discus* (BAH10148.1), *H. rufescens* (CAA53028.1), *Crassostrea gigus* (ARX70262.1), *Mizuhopecten yessoensis* (ACF22883.1), *Scylla serrata* (ABS12233.1), *Pennahia argentata* (BAB20881.1), *Rattus norvegicus* (AAA42263.1), *Gallus gallus* (NP_990732.1), and *Homo sapiens* (AAT68295.1).

Motifs of Hdh-TPM were analogously expressed when they were compared with different TPM protein sequences of other vertebrate and invertebrate species. A total of eight motifs were recognized in Hdh-TPM. Similarly, eight motifs were recognized in the other compared TPM protein sequences. The C-terminal motif of invertebrates was homologous, but heterologous C-terminal motifs were found in vertebrates (Figure 2).

**Figure 3:**
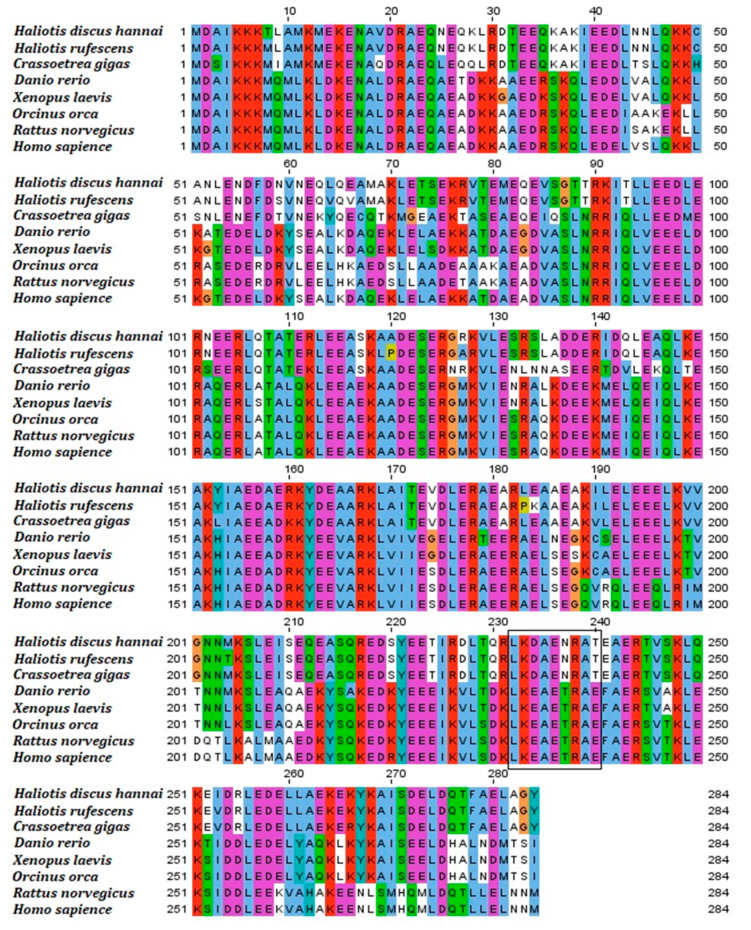
The multiple sequence alignment of Hdh-TPM from deduced amino acid sequences of *H. discus hannai* (OM937906), *H. rufescens* (X75218.1), *H. discus discus* (AB444939.1), *Crassostrea gigus* (AB444943.1), *Mizuhopecten yessoensis* (AB004636.1), *Scylla serrata* (EF672351.1), *Pennahia argentata* (AB045645.2), *Mus musculus* (AAI32038.1), *Gallus gallus* (AAA49112.1), and *Homo sapiens* (AAA36771.1). Tropomyosin signature is indicated by a black outlined box.

The multiple sequence alignment revealed that 101 residues of the deduced Hdh-TPM amino acid sequence were conserved when aligned with TPM of *H. discus hannai*, *H. rufescens*, *Crassostrea gigas*, *Danio rerio*, *Xenopus laevis*, *Orcinus orca*, *Rattus norvegicus*, and *Homo sapiens* (Figure 3). The signature motifs of the invertebrates were the same; however, when compared with vertebrates, the signature motifs differed. The third, sixth, and ninth positions of the signature motif possessed glutamic acid (E), threonine (T), and glutamic acid (E), respectively, in vertebrates, but possessed aspartic acid (D), asparagine (N), and threonine (T), respectively, in invertebrates. The N-terminal region of Hdh-TPM was more conserved than the C-terminal region for vertebrates and invertebrates.

### 3.2. Structure of the Hdh-TPM Protein

**Figure 4:**
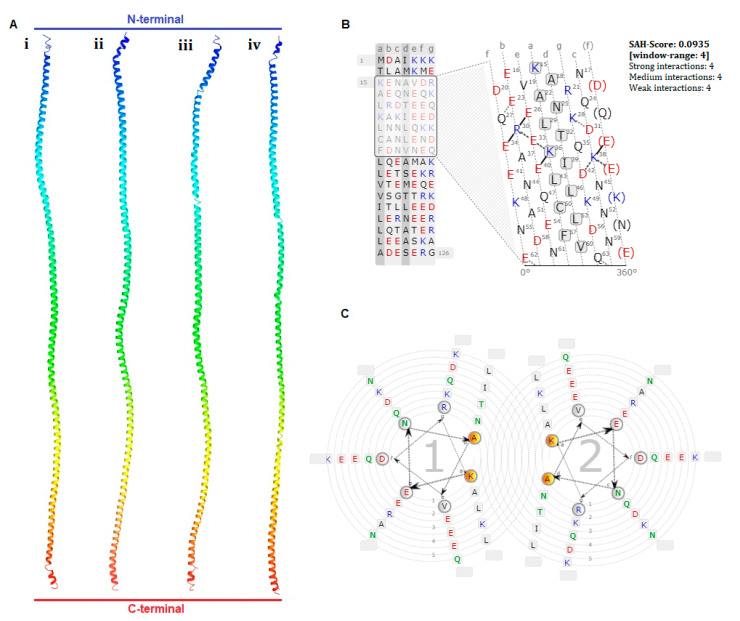
(**A**) Three-dimensional structure prediction of amino acid sequence from four abalone species (i) *H. discus hannai*, (ii), *H. asisina*, (iii) *H. discus discus* and (iv) *H. rufescens*. (**B**) A single α helical (SAH) interaction in the protein heptad net view. (**C**) A coiled-coil wheel view of the Hdh-TPM protein.

The 3D structure of Hdh-TPM consisted of the secondary protein structure, a long coiled-coil α-helical structure (Figure 4A(i)). Hdh-TPM showed an almost similar 3D structure when compared with the TPM of *H. asisina* (Figure 4A(ii)), *H. discus discus* (Figure 4A(iii)), and *H. rufescens* (Figure 4A(iv)). The heptad repeat, a common structure of coiled-coil proteins predicted in the Hdh-TPM sequence, had four strong amino acid interactions, E–R (26 and 30), R–E (30 and 34), E–K (34 and 38), and K–E (36 and 40), with a single α helix (SAH) score of 0.0935 between the residues 15 and 63 in the heptad net view (Figure 4B). The heptad wheel view formed a dimer during the coiled-coil probability prediction (Figure 4C).

### 3.3. Properties and Gene Ontology of the Hdh-TPM Amino Acid Sequence

**Figure 5:**
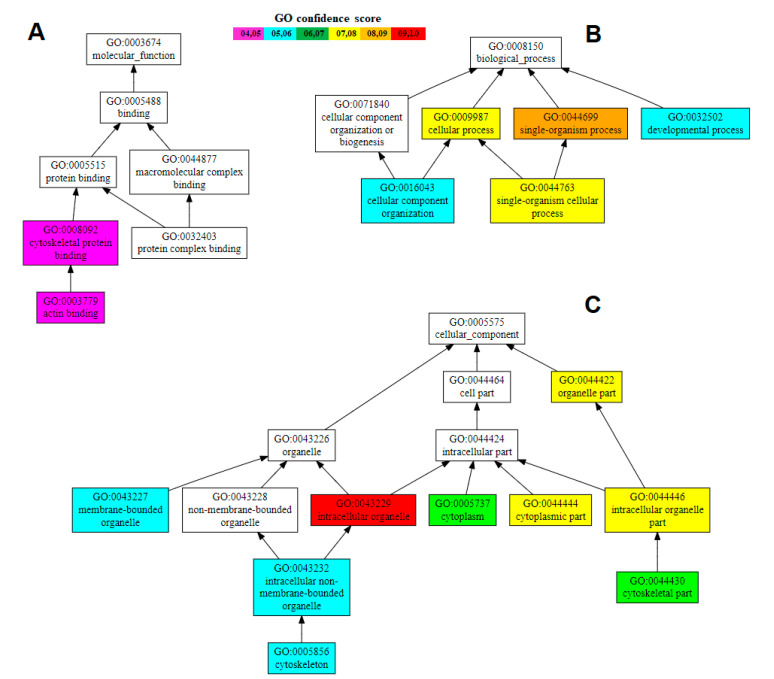
Gene ontology based on the Hdh-TPM protein sequence: (**A**) Molecular function indicating actin-binding cytoskeletal protein. (**B**) Biological processes, including single-organism cellular process through cellular component organization and development. (**C**) Cellular component indicating intracellular organelle, especially the cytoskeleton.

The theoretical molecular weight and pI of the Hdh-TPM protein were 32.86433 kDa and 4.75, respectively. The gene ontology (GO) analysis using the C-I-TASSER server predicted that the Hdh-TPM protein acted on the following biological process: single-organism cellular process (GO: 0044699) with a C-scoreGO of 0.78; the following cellular component: intracellular organelle (cytoskeleton part) (GO: 0043229) with a C-scoreGO of 1.00; and the following molecular function: actin-binding cytoskeletal protein (GO: 0008092) with a C-scoreGO of 0.48 (Figure 5).

### 3.4. Orthology Assessment

**Figure 6:**
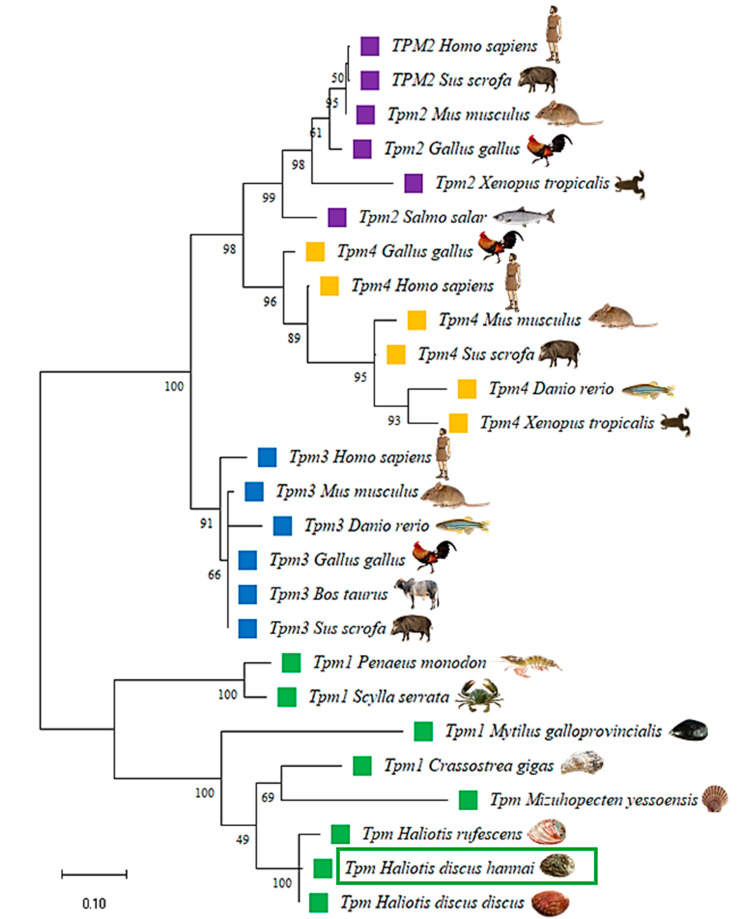
Phylogenetic tree constructed using the maximum likelihood method with a bootstrap value of 1000 after clustalW alignment based on amino acid residues of different isoforms of tropomyosin. The following sequences with their protein ID were used to construct the phylogenetic tree: TPM/TPM1 of *H. discus discus* (BAH10148.1), *H. discus hannai* (AAR45321.1), *H. rufescens* (CAA53028.1), *Crassostrea gigus* (ARX70262.1), *Mizuhopecten yessoensis* (BAA20455.1), *Mytilus galloprovincialis* (VDI44733.1), *Scylla serrata* (ABS12233.1), and *Penaeus monodon* (ADV17340.1); TPM3 of *Sus scrofa* (NP_001001632.1), *Bos taurus* (NP_001011674.1), *Gallus gallus* (ATC20309.1), *Mus musculus* (AAG38596.1), *Homo sapiens* (ATC20307.1), and *Danio rerio* (NP_991239.1); TPM4 of *Danio rerio* (AAH53144.1), *Xenopus tropicalis* (CAJ82227.1), *Sus scrofa* (NP_999500.1), *Mus musculus* (AAH23701.1), *Gallus gallus* (XP_046789950.1), and *Homo sapiens* (NP_001138632.1); TPM2 of *Danio rerio* (XP_005155551.1), *Xenopus tropicalis* (NP_001025587.1), *Gallus gallus* (XP_046790843.1), *Sus scrofa* (NP_001123419.1), *Homo sapiens* (KAI2552574.1), and *Mus musculus* (NP_001264805.1).

An unrooted phylogenetic tree was constructed using the maximum likelihood method based on the amino acid sequences of TPM proteins from various species, and showed four major groups: TPM, TPM2, TPM3, and TPM4 (Figure 6). The Hdh-TPM fitted with the TPM group and clustered with its phylogenetically closest matches, TPM of *H. discus discus* and *H. asinina*.

### 3.5. Expression of Hdh-TPM in Different Tissues

**Figure 7:**
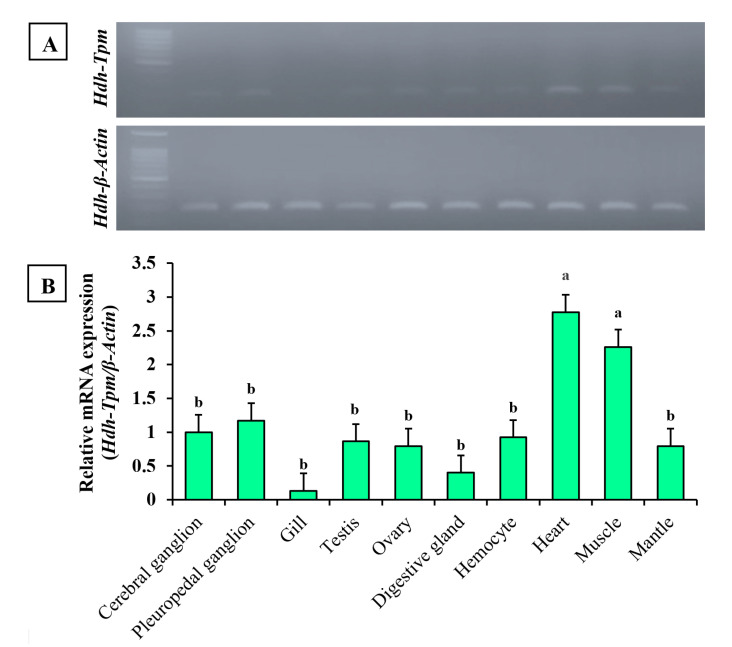
Expression of *Hdh-TPM* mRNA in different tissues of Pacific abalone *H. discus hannai*: (**A**) semiquantitative expression; (**B**) quantitative expression. Significantly different levels (*p* < 0.05) are denoted by different letters.

In the tissue distribution analysis, the *Hdh-TPM* gene was highly expressed in the heart and muscle tissue of Pacific abalone, *H. discus hannai*. The expression was found to be very weak in gill tissue (Figure 7A). Similar expression was found during qRT-PCR analysis. The expression of *Hdh-TPM* was significantly higher in the heart and muscle (*p* < 0.05) than in the pleuropedal ganglion, branchial ganglion, testis, ovary, digestive gland, and mantle tissues (Figure 7B). Significantly lower expression was found in the gill (*p* < 0.05). Supporting data were also found from the semiquantitative RT-PCR expression analysis.

### 3.6. Hdh-TPM Expression at Different Stages of Embryonic and Larval Development (ELD)

**Figure 8:**
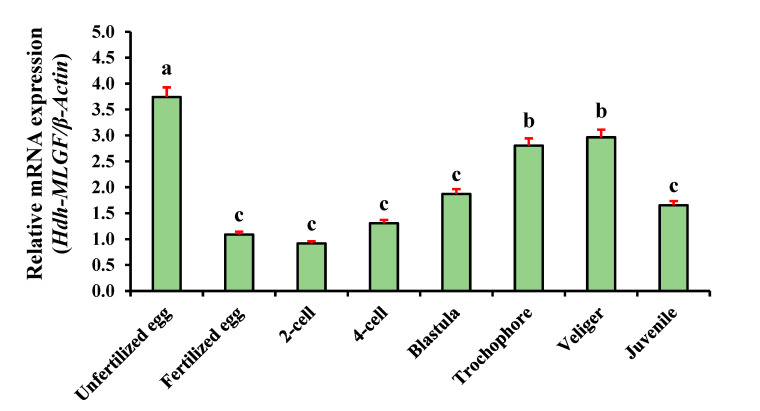
Expression of *Hdh-TPM* mRNA in embryonic and larval developmental stages of Pacific abalone. Significantly different levels (*p* < 0.05) are denoted by different letters.

The results of the expression analysis revealed that throughout the embryonic and larval development (ELD) stages, *Hdh-TPM* was expressed ubiquitously. *Hdh-TPM* mRNA was highly expressed in the unfertilized eggs, but the expression level was significantly reduced immediately after fertilization (fertilized eggs). However, there was some increased level of expression found during the cell division phases. The expression level was lowest in the two-cell stage compared with other examined stages. After fertilization, veliger showed the maximum expression followed by the first larval stage (trochophore), blastula, juvenile, four-cell, and fertilized egg stages (Figure 8).

### 3.7. Expression of Hdh-TPM in Different Growth Types

**Figure 9:**
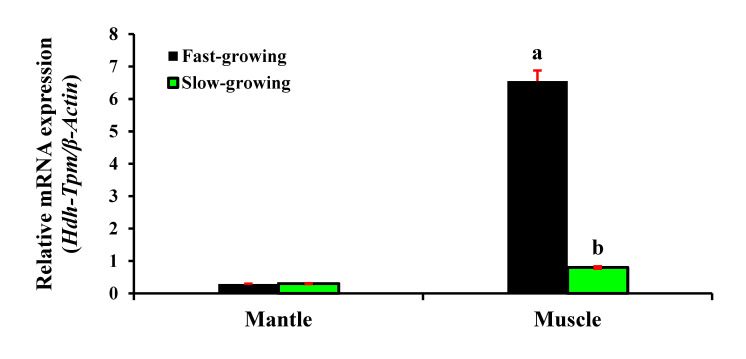
Expression of *Hdh-TPM* in the mantle and muscle tissues of different-growth-type Pacific abalones, *H. discus hannai*. Significantly different levels (*p* < 0.05) are denoted by different letters.

The *Hdh-TPM* were differentially expressed in the fast- and slow-growing abalones. The relative mRNA expression level of *Hdh-TPM* in the muscle tissue of the fast-growing group showed higher expression compared with the slow-growing group during the study period (Figure 9). In contrast, the expression of *Hdh-TPM* in the mantle tissue of fast-growing and slow-growing groups was similar and significantly lower than that of the muscle tissue.

### 3.8. Expression of Hdh-TPM at Different Heat Stress Conditions

**Figure 10:**
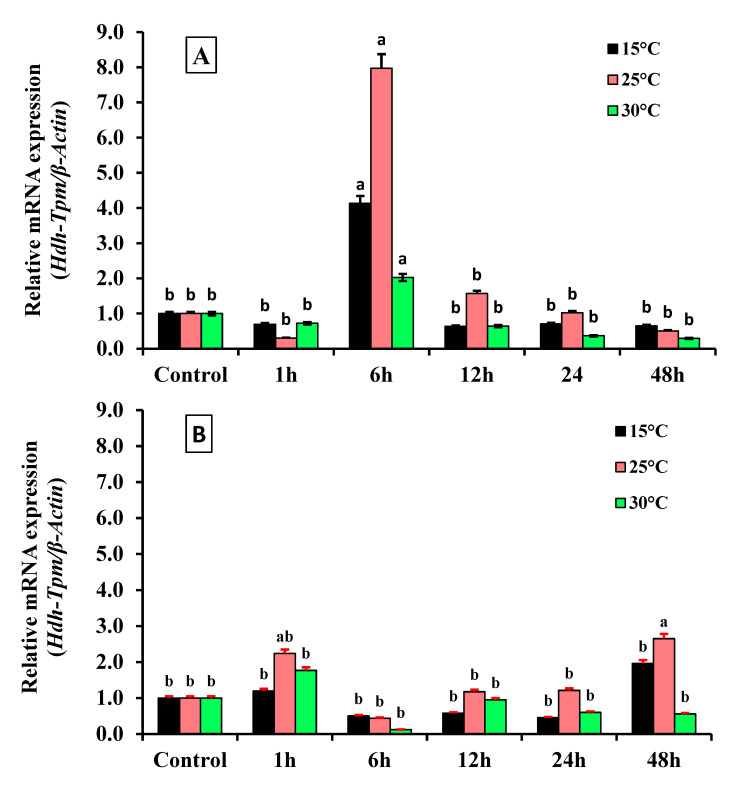
Expression of *Hdh-TPM* mRNA at different heat stress conditions: (**A**) Muscle tissue and (**B**) mantle tissue of Pacific abalone. Significantly different levels (*p* < 0.05) are denoted by different letters.

During heat stress, the expression of *Hdh-TPM* in the mantle and muscle was quite different. Immediately after the heat treatment, the expression was reduced in the muscle tissue compared with the control, but was not significant. The expression of *Hdh-TPM* was found to be significantly higher after six hours of heat stress for all the tested temperatures (15, 25, and 30 °C) compared with controls and the other time points. Exposure to 25 °C caused the highest upregulation of Hdh-TPM, which doubled and tripled the ones induced by, respectively, the 15 °C and 30 °C treatments. After 12 h, the expression of *Hdh-TPM* at 15 °C was reduced and stable for all the time points up to 48 h. The expression of *Hdh-TPM* after six hours at 25 and 30 °C reduced gradually for all the subsequent time points tested. The minimum expression was found to be after 48 h of heat stress for all tested temperatures (Figure 10A). In the case of the mantle, the expression of *Hdh-TPM* was almost different to that of the muscle tissue. The highest expression was observed after 48 h, and the lowest expression among the tested temperatures was found at six hours (Figure 10B).

### 3.9. Expression of Hdh-TPM in Different Seasons

**Figure 11:**
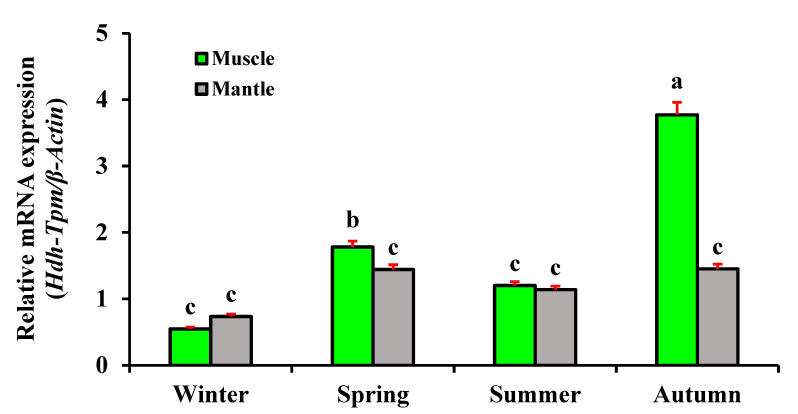
Expression of *Hdh-TPM* according to the season in mantle and muscle tissues of Pacific abalone *H. discus hannai*. Significantly different levels (*p* < 0.05) are denoted by different letters.

*Hdh-TPM* was differentially expressed according to the seasons. In the winter, the expression of *Hdh-TPM* in the muscle tissue was significantly lower than in other seasons. The expression of *Hdh-TPM* was significantly increased in the spring, and the maximum expression was observed in the autumn (Figure 11). The expression of *Hdh-TPM* in the mantle tissue changed but was not significant. On average, the highest expression of *Hdh-TPM* was in the autumn for the muscle and mantle tissue.

### 3.10. Expression of Hdh-TPM in Starvation Conditions

**Figure 12:**
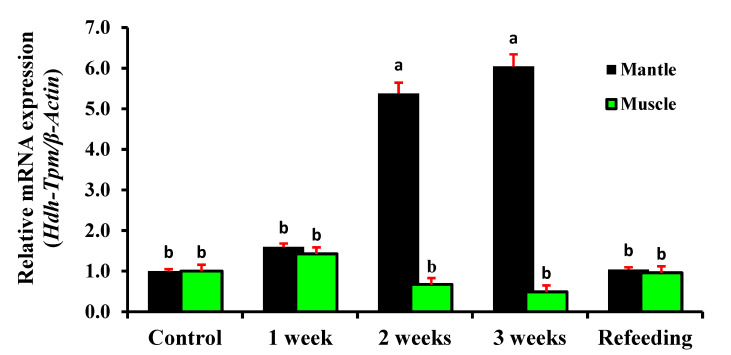
Expression of *Hdh-TPM* in the mantle and muscle of starved Pacific abalone *H. discus hannai.* Significantly different levels (*p* < 0.05) are denoted by different letters.

During starvation (nutritional stress) conditions, significant differences were observed in the expression of *Hdh-TPM* in the mantle tissue. The expression level of *Hdh-TPM* was increased with starvation until the refeeding, and the maximum expression was found to be after three weeks of starvation (Figure 12). In contrast, the expression level of *Hdh-TPM* in the muscle was increased after the first week of starvation but then decreased until the refeeding. In the muscle, the lowest expression was recorded in the third week but was not significant. However, after refeeding, the expression of *Hdh-TPM* in the mantle and muscle was similar to the control.

## 4. Discussion

TPMs are a large family of actin-binding filament proteins extensively found in vertebrates and invertebrates [30]. TPM first came to prominence because of its role in regulating muscle contraction. Together with the other contractile proteins, actin, troponin, and myosin, TPM regulates the contraction and relaxation of muscle and non-muscle cells [31]. At present, four TPM genes (TPM/TPM1, TPM2, TPM3, and TPM4) have been discovered in mammalian cells, with four variants (α, β, γ, and δ) and at least 28 isoforms [32] with specific functions, including actin cytoskeletal functions such as cell motility, cell division, intracellular trafficking, and the maintenance of cell shape [33]. TPM/TPM1 and its different isoforms are reported to be involved in cell motility [34], muscle contraction [35], myofibril organization, myocardial contraction, cardiac development [36,37], stabilization of stress fibers [38], filament protection [39], formation of filopodia [40], the rescue of transformed cells [41], heart muscle contraction, tissue-specific development [42], hypertrophic cardiomyopathy, regulation of cystic fibrosis transmembrane conductance [43], focal adhesion formation, and lamellipodial persistence [44].

Successfully cloned *Hdh-TPM* from the muscle tissue of Pacific abalone had a high aliphatic index, indicating a thermophilic protein. The main features of this protein are the presence of a long α-helical coiled-coil TPM domain and a C-terminus TPM signature. Generally, coiled-coils are involved in a much wider range of biological functions, including signal transduction, regulation of gene expression, oligomerization, and transport of other molecules [45]. Since the coiled-coil α helices of TPM are reported to regulate muscle contraction by cooperatively exposing and blocking the myosin-binding sites of actin, *Hdh-TPM* might be involved in muscle contraction. The coiled-coil signature motif also performs a role in steric regulation [7] and is conserved in all proteins (TPM/TPM1, TPM2, TPM3, and TPM4) of the TPM family. The volatile positions of the TPM signature are fixed by aspartic acid and threonine in Hdh-TPM. The sequence also contains a C-terminus spectrin repeat domain, common to TPM proteins (actin-binding), which may be involved in cytoskeletal structure [46].

The tissue-specific mRNA expression analysis showed higher expression in the heart and muscle tissues. These three organs are contractile organs, indicating the contractile function of *Hdh-TPM* in Pacific abalone. In contrast, a lower expression was found in the non-contractile gill, suggesting that *Hdh-TPM* has no function regarding contractility in this organ. Some researchers have reported that TPM functions in ovarian contraction [12]; however, the expression of *Hdh-TPM* was relatively low in the ovary. The probable reason for this lower expression may be related to the ovarian development stage.

*Hdh-TPM* was dynamically expressed throughout the ELD stages of Pacific abalone. Although the present study found that the highest expression was in the unfertilized eggs, the role of *Hdh-TPM* at this stage remains unclear [47]. The higher expression of *Hdh-TPM* in trochophore and veliger larvae suggests that it may play a role in complex cellular processes, including muscle development. In the small abalone, *Haliotis diversicolor*, the encapsulated retractor muscle appears in the trochophore stage and is enlarged in the veliger stage [48]. In abalone, *Hdh-TPM* may be involved in these activities because the GO analysis indicated that it was involved in the cellular component organization and development process, and the abalone retractor muscle appears in the trochophore stage.

The higher expression of TPM in contractile tissue indicates a higher actin-binding activity, and the lower expression indicates a low actin-binding activity regulating contractility. The expression of *Hdh-TPM* was found to be upregulated in the mantle and muscle tissues of fast-growing abalones. However, the expression was significantly higher in the muscle compared with the mantle. Since the tissue was collected from fast-growing and slow-growing abalones of the same age from identical parents and cultured in the same environmental conditions, including food availability, there was less chance of influencing their growth pattern by these factors. Therefore, the growth variability in the abalone muscle was most likely due to the activation of a growth inhibitory gene, myostatin, which is somewhat regulated during muscle contraction via the actin-binding activity of Hdh-TPM. Skeletal muscles are some of the most dynamic tissues involved in voluntary contractions [49]. The higher expression of *Hdh-TPM* indicated a higher actin-binding activity and a higher muscle contraction rate. The information regarding muscle contraction or the effects of tropomyosin on fast- and slow-growing animals is scarce. This is the preliminary study about the functional activity of tropomyosin in different-growth-type animals.

The temperature dependence of physiological processes in marine ectoderms is well recognized [50]. Hdh-TPM is a thermophilic protein, and the expression of this gene can change depending on the temperature. The present study found differential expression of *Hdh-TPM* in the muscle and mantle at different ambient temperatures. In the muscle tissue, the comparatively lower *Hdh-TPM* mRNA expression after one hour of heat stress is indicative of a reduction in actin-binding activity and muscle contraction. Although the underlying cause of the decreased *Hdh-TPM* mRNA expression after one hour of acclimation to the controlled temperature is unknown, it may be related to sudden heat stress and stress induced by the animal handling during the experimental setup. The optimum muscle contraction for jack mackerel was found to be 18 °C, and the contraction rate was found to be stable at temperatures ranging from 15 to 22 °C [13]. Additionally, the speed of muscle contraction was found to be slower at lower temperatures than at higher temperatures [13]. The lower expression at 15 °C (except six hours after thermal stress) indicated a lower actin-binding activity and negatively affected muscle contraction. Generally, muscle contractility increases with increasing water temperatures [51] within the tolerance range. In the case of abalone, increasing water temperatures increase the metabolic rate; however, temperatures above 20 °C reduce the feeding rate due to heat stress tension [18]. Thus, the extensively higher expression of *Hdh-TPM* at 25 °C after six hours of stress treatment may indicate overexpression. Overexpression of *Hdh-TPM* at 30 °C may occur earlier than six hours (possibly after 3–4 h of heat stress treatment); it began to decrease after reaching the peak.

In response to seasonal change, *Hdh-TPM* showed differential expression in the mantle and muscle tissues. Seasonal changes are temperature-dependent, with winter temperatures ranging from 3 to 8 °C, spring and autumn temperatures ranging from 13 to 18 °C, and summer temperatures ranging from 23 to 28 °C in Korea [50]. Generally, optimum muscle contraction is found during optimum growth conditions, which are primarily influenced by temperature. During the autumn and spring seasons, temperatures were within the suitable range for optimum growth, and the maximum expression of *Hdh-TPM* was observed, indicating muscle contraction in these seasons.

Starvation is one of the common nutritional stresses that can affect abalone body physiology, by influencing the expression of several genes [52]. The present study found upregulated expression of *Hdh-TPM* during the first week of starvation in the mantle and muscle tissues. In crickets, upregulated expression of TPM was also previously reported during short-term starvation [52]. However, after the first week, the expression of *Hdh-TPM* significantly increased in the mantle and decreased in the muscle (not significant) until refeeding. The reason for the significantly higher expression in the mantle is unknown; however, the lower expression in the muscle may be related to reduced contraction due to a decreased Ca^2+^ supply that mainly comes from dietary sources [53]. Short-term starvation does not interfere with normal muscle physiology, including normal production of myokines such as decorin, that are released during muscle contraction [54]. Decorin directly binds to myostatin, a potent inhibitor of muscle growth. At the onset of starvation, animals convert fat into an energy (ATP) source, which may not influence muscle growth. However, skeletal muscle protein breaks down during long-term starvation through proteolysis and hampers muscle contraction and decorin release. Energy (ATP) from food is needed for the actin-binding activity; however, during starvation, ATP comes from fat decomposition and later from protein breakdown, which reduces the expression of *Hdh-TPM* and muscle contraction rate and, thus, negatively affects muscle growth.

## 5. Conclusions

TPM is a well-known muscle protein that largely controls muscle contraction by binding to actin. Coiled-coil α-helical structures of the Hdh-TPM protein sequence may be involved in this activity. GO and the functional domain of the cloned *Hdh-TPM* also indicated a contractile function that might play a role in muscle growth. The expression of *Hdh-TPM* in early development indicates that *Hdh-TPM* may be involved in ELD, especially in the development of the retractor muscle. In response to thermal stress and seasonal variation, the expression of *Hdh-TPM* also indicated contractile activity. Reduced expression of *Hdh-TPM* in Pacific abalone muscle tissue during starvation was also indicative of lower muscle contraction because the Ca^2+^ available in food required for contraction was not supplied. Altogether, the findings are indicative of a potential function of TPM in muscle contraction and, thus, growth in the abalone *Haliotis discus hannai*.

## Figures and Tables

**Table 1:** List of different primers used for cDNA synthesis, cloning, and expression analysis in this study.

Primer Name	Nucleotide Sequences	Purpose
Oligo dT (OdT)	GGCCACGCGTCGACTAGTACTTTTTTTTTTTTTTTTT	cDNA synthesis
Oligo dT adapter	GGCCACGCGTCGACTAGTAC	
TPM Fw	CAAACTACAGCCATGGATGC	Fragment PCR
TPM Rv	GAGATGCCTCTTGTTCACTG	Fragment PCR
TPM 5′	GATTACGCCAAGCTTGTGACAGAGATGGAACAAGAGGTGAGTG	5′ RACE PCR
TPM 3′	GATTACGCCAAGCTT GAGAACCGGGCAACTGAGGCTGAAAG	3′ RACE PCR
TPM-ORF-Fw	CAAACTACAGCCATGGATGC	Full length PCR
TPM-ORF-Rv	GCCATATGGATTTTGTAGGC	Full length PCR
TPM-qRT-Fw	CAGTGAACAAGAGGCATCTC	qPCR
TPM-qRT-Rv	CGTCACTAATGGCCTTGTAC	qPCR
Hdh-β-Actin-Fw	CCGTGAAAAGATGACCCAGA	qRT-PCR
Hdh-β-Actin-Rv	TACGACCGGAAGCGTACAGA	

## Data Availability

All data generated in this study are included in this article.

## References

[1] Kalyva A., Schmidtmann A., Geeves M.A. (2012). In vitro formation and characterization of the skeletal muscle α·β Tropomyosin heterodimers. Biochemistry.

[2] Gunning P.W., Ghoshdastider U., Whitaker S., Popp D., Robinson R.C. (2015). The evolution of compositionally and functionally distinct actin filaments. J. Cell Sci..

[3] Behrmann E., Muller M., Penczek P.A., Mannherz H.G., Manstein D.J., Raunser S. (2012). Structure of the rigor actin-Tropomyosin-myosin complex. Cell.

[4] Barua B., Winkelmann D.A., White H.D., Hitchcock-DeGregori S.E. (2012). Regulation of actin-myosin interaction by conserved periodic sites of Tropomyosin. Proc. Natl. Acad. Sci. USA.

[5] Loong C.K., Badr M.A., Chase P.B. (2012). Tropomyosin flexural rigidity and single Ca^2+^ regulatory unit dynamics: Implications for cooperative regulation of cardiac muscle contraction and cardiomyocyte hypertrophy. Front. Physiol..

[6] Pavadai E., Lehman W., Rynkiewicz M.J. (2020). Protein-Protein Docking Reveals Dynamic interactions of Tropomyosin on actin filaments. Biophys. J..

[7] Lehman W., Rynkiewicz M.J., Moore J.R. (2020). A new twist on tropomyosin binding to actin filaments: Perspectives on thin filament function, assembly and biomechanics. J. Muscle Res. Cell Motil..

[8] Lee J.H., Jun H.-S. (2019). Role of Myokines in Regulating Skeletal Muscle Mass and Function. Front. Physiol..

[9] Douglas J., Pearson S., Ross A., McGuigan M. (2017). Chronic adaptations to eccentric training: A systematic review. Sports Med..

[10] Schiaffino S., Dyar K.A., Ciciliot S., Blaauw B., Sandri M. (2013). Mechanisms regulating skeletal muscle growth and atrophy. FEBS J..

[11] Li X.Y., Lin Y.S., Zhang H.W. (2012). Phylogenetic analysis and expression patterns of Tropomyosin in amphioxus. Dongwuxue Yanjiu.

[12] Ono K., Ono S. (2004). Tropomyosin and troponin are required for ovarian contraction in the Caenorhabditis elegans reproductive system. Mol. Biol. Cell..

[13] Kang H.Y., Lee Y.-J., Song W.-Y., Kim T.-I., Lee W.-C., Kim T.Y., Kang C.-K. (2019). Physiological responses of the abalone *Haliotis discus hannai* to daily and seasonal temperature variations. Sci. Rep..

[14] Brooks G.A., Hittleman K.J., Faulkner J.A., Beyer R.E. (1971). Tissue temperature and whole-animal oxygen consumption after exercise. Am. J. Physiol..

[15] Allen D.G. (2009). Fatigue in working muscles. J. Appl. Physiol..

[16] Locke M., Celotti C. (2014). The effect of heat stress on skeletal muscle contractile properties. Cell Stress Chaperones.

[17] Saunderson E.A., Spiers H., Mifsud K.R., Gutierrez-Mecinas M., Trollope A.F., Shaikh A., Mill J., Reul J.M. (2016). Stress-induced gene expression and behavior are controlled by DNA methylation and methyl donor availability in the dentate gyrus. Proc. Natl. Acad. Sci. USA.

[18] Britz P.J., Hecht T., Mangold S. (1997). Effect of temperature on growth, feed consumption and nutritional indices of *Haliotis midae* fed a formulated diet. Aquaculture.

[19] Sukhan Z.P., Hossen S., Cho Y., Lee W.K., Kho K.H. (2022). Hdh-Tektin-4 Regulates Motility of Fresh and Cryopreserved Sperm in Pacific Abalone, *Haliotis discus hannai*. Front. Cell Dev. Biol..

[20] Hsu T.H., Gwo J.C. (2017). Genetic diversity and stock identification of small abalone (*Haliotis diversicolor*) in Taiwan and Japan. PLoS ONE.

[21] Young M.A., Treml E.A., Beher J., Fredle M., Gorfine H., Miller A.D., Swearer S.E., Ierodiaconou D. (2020). Using species distribution models to assess the long-term impacts of changing oceanographic conditions on abalone density in southeast Australia. Ecography.

[22] Kyeong D., Kim J., Shin Y., Subramaniyam S., Kang B.-C., Shin E.-H., Park E.H., Noh E.S., Kim Y.-O., Park J.Y. (2020). Expression of Heat Shock Proteins in Thermally Challenged Pacific Abalone *Haliotis discus hannai*. Genes.

[23] Hossen S., Sukhan Z.P., Cho Y., Kho K.H. (2021). Effects of Cryopreservation on Gene Expression and Post Thaw Sperm Quality of Pacific Abalone, *Haliotis discus hannai*. Front. Mar. Sci..

[24] Park C.-J., Kim S.Y. (2013). Abalone aquaculture in Korea. J. Shellfish Res..

[25] Xu F., Gao T., Liu X. (2020). Metabolomics Adaptation of Juvenile Pacific Abalone *Haliotis discus hannai* to Heat Stress. Sci. Rep..

[26] Sharker M.R., Kim S.C., Hossen S., Sumi K.R., Choi S.K., Choi K.S., Kho K.H. (2021). Carbonic Anhydrase in Pacific Abalone *Haliotis discus hannai*: Characterization, Expression, and Role in Biomineralization. Front. Mol. Biosci..

[27] Huang J., Luo X., Huang M., Liu G., You W., Ke C. (2018). Identification and characteristics of muscle growth-related microRNA in the Pacific abalone, *Haliotis discus hannai*. BMC Genom..

[28] Mohammadabadi M., Bordbar F., Jensen J., Du M., Guo W. (2021). Key Genes Regulating Skeletal Muscle Development and Growth in Farm Animals. Animals.

[29] Hanif M.A., Hossen S., Cho Y., Sukhan Z.P., Choi C.Y., Kho K.H. (2022). Characterization and Expression Analysis of Mollusk-like Growth Factor: A Secreted Protein Involved in Pacific Abalone Embryonic and Larval Development. Biology.

[30] Meiring J.C.M., Bryce N.S., Wang Y., Taft M.H., Manstein D.J., Lau S.L., Steer J., Hardeman E.C., Gunning P.W. (2018). Co-polymers of Actin and Tropomyosin Account for a Major Fraction of the Human Actin Cytoskeleton. Curr. Biol..

[31] Choi Y.M., Kim B.C. (2009). Muscle fiber characteristics, myofibrillar protein isoforms, and meat quality. Livest. Sci..

[32] Manstein D.J., Meiring J.C.M., Hardeman E.C., Gunning P.W. (2020). Actin–Tropomyosin distribution in non-muscle cells. J. Muscle Res. Cell Motil..

[33] Gunning P., O’Neill G., Hardeman E. (2008). Tropomyosin-based regulation of the actin cytoskeleton in time and space. Physiol. Rev..

[34] Hillberg L., Zhao-Rathje L.S., Nyakern-Meazza M., Helfand B., Goldman R.D., Schutt C.E., Lindberg U. (2006). Tropomyosins are present in lamellipodia of motile cells. Eur. J. Cell Biol..

[35] Smith D.A. (2018). The Sliding-Filament Theory of Muscle Contraction.

[36] England J., Granados-Riveron J., Polo-Parada L., Kuriakose D., Moore C., Brook J.D., Rutland C.S., Setchfield K., Gell C., Ghosh T.K. (2017). Tropomyosin 1: Multiple roles in the developing heart and in the formation of congenital heart defects. J. Mol. Cell. Cardiol..

[37] Cao J., Routh A.L., Kuyumcu-Martinez M.N. (2021). Nanopore sequencing reveals full-length Tropomyosin 1 isoforms and their regulation by RNA-binding proteins during rat heart development. J. Cell. Mol. Med..

[38] Tojkander S., Gateva G., Schevzov G., Hotulainen P., Naumanen P., Martin C., Gunning P.W., Lappalainen P. (2011). A molecular pathway for myosin II recruitment to stress fibers. Curr. Biol..

[39] Gateva G., Kremneva E., Reindl T., Kotila T., Kogan K., Gressin L., Gunning P.W., Manstein D.J., Michelot A., Lappalainen P. (2017). Tropomyosin isoforms specify functionally distinct actin filament populations in vitro. Curr. Biol..

[40] Creed S.J., Desouza M., Bamburg J.R., Gunning P., Stehn J. (2011). Tropomyosin isoform 3 promotes the formation of filopodia by regulating the recruitment of actin-binding proteins to actin filaments. Exper. Cell. Res..

[41] Gimona M., Kazzaz J.A., Helfman D.M. (1996). Forced expression of tropomyosin 2 or 3 in v-ki-ras-transformed fibroblasts results in distinct phenotypic effects. Proc. Natl. Acad. Sci. USA.

[42] Gooding C., Smith C.W. (2008). Tropomyosin exons as models for alternative splicing. Adv. Exp. Med. Biol..

[43] Dalby-Payne J.R., O’Loughlin E.V., Gunning P. (2003). Polarization of specific tropomyosin isoforms in gastrointestinal epithelial cells and their impact on cftr at the apical surface. Mol. Biol. Cell..

[44] Brayford S., Bryce N.S., Schevzov G., Haynes E.M., Bear J.E., Hardeman E.C., Gunning P.W. (2016). Tropomyosin promotes lamellipodial persistence by collaborating with arp2/3 at the leading edge. Curr. Biol..

[45] Szczepaniak K., Bukala A., da Silva Neto A.M., Ludwiczak J., Dunin-Horkawicz S. (2020). A library of coiled-coil domains: From regular bundles to peculiar twists. Bioinformatics.

[46] Djinovic-Carugo K., Gautel M., Ylanne J., Young P. (2002). The spectrin repeat: A structural platform for cytoskeletal protein assemblies. FEBS Lett..

[47] Ishimoda-Takagi T. (1979). Localization of tropomyosin in sea urchin eggs. Exp. Cell Res..

[48] Di G., Kong X., Miao X., Zhang Y., Huang M., Gu Y., You W., Zhang J., Ke C. (2017). Proteomic analysis of trochophore and veliger larvae development in the small abalone Haliotis diversicolor. BMC Genom..

[49] Frontera W.R., Ochala J. (2015). Skeletal muscle: A brief review of structure and function. Calcif. Tissue Int..

[50] Nofrizal, Ramdhani F., Arimoto T. (2020). Temperature effect on the maximum swimming speed of jack mackerel *Trachurus japonicus* through muscle contraction monitoring. Anim. Behav. Biometeorol..

[51] Yanase K., Eayrs S., Arimoto T. (2007). Influence of water temperature and fish length on the maximum swimming speed of sand flathead, *Platychephalus bassensis*: Implication for trawl selectivity. Fish. Res..

[52] Kwon K., Yoo B.-K., Ko Y., Choi J.-Y., Kwon O.-Y., Kim S.-W. (2018). Effect of starvation on expression of troponin complex genes and ER stress associated genes in skeletal muscles. Biomed Res..

[53] Shkembi B., Huppertz T. (2021). Calcium Absorption from Food Products: Food Matrix Effects. Nutrients.

[54] Kanzleiter T., Rath M., Görgens S.W., Jensen J., Tangen D.S., Kolnes A.J., Kolnes K.J., Lee S., Eckel J., Schürmann A. (2014). The myokine decorin is regulated by contraction and involved in muscle hypertrophy. Biochem. Biophys. Res. Commun..

